# Diversification of plant *SUPPRESSOR OF MAX2 1* (*SMAX1*)-like genes and genome-wide identification and characterization of cotton *SMXL* gene family

**DOI:** 10.1186/s12870-023-04421-6

**Published:** 2023-09-11

**Authors:** Bin Ma, Jianbo Zhu, Xianzhong Huang

**Affiliations:** 1 College of Life Sciences, Shihezi University, Shihezi, 832003 China; 2 Center for Crop Biotechnology, College of Agriculture, Anhui Science and Technology University, Fengyang, 233100 China

**Keywords:** Plant architecture, SMAX1, SMXL, Strigolactones, Branching

## Abstract

**Background:**

Strigolactones (SLs) are a recently discovered class of plant hormones. SUPPRESSOR OF MAX2 1 (SMAX1)-like proteins, key component of the SL signaling pathway, have been studied extensively for their roles in regulating plant growth and development, such as plant branching. However, systematic identification and functional characterization of *SMXL* genes in cotton (*Gossypium* sp.), an important fiber and oil crop, has rarely been conducted.

**Results:**

We identified 210 *SMXL* genes from 21 plant genomes and examined their evolutionary relationships. The structural characteristics of the *SMXL* genes and their encoded proteins exhibited both consistency and diversity. All plant SMXL proteins possess a conserved Clp-N domain, P-loop NTPase, and EAR motif. We identified 63 *SMXL* genes in cotton and classified these into four evolutionary branches. Gene expression analysis revealed tissue-specific expression patterns of *GhSMXL* genes, with some upregulated in response to GR24 treatment. Protein co-expression network analysis showed that GhSMXL6, GhSMXL7-1, and GhSMXL7-2 mainly interact with proteins functioning in growth and development, while virus-induced gene silencing revealed that *GhSMAX1-1* and *GhSMAX1-2* suppress the growth and development of axillary buds.

**Conclusions:**

*SMXL* gene family members show evolutionary diversification through the green plant lineage. *GhSMXL6*/*7–1*/*7–2* genes play critical roles in the SL signaling pathway, while *GhSMXL1-1* and *GhSMXL1-2* function redundantly in growth of axillary buds. Characterization of the cotton *SMXL* gene family provides new insights into their roles in responding to SL signals and in plant growth and development. Genes identified in this study could be used as the candidate genes for improvement of plant architecture and crop yield.

**Supplementary Information:**

The online version contains supplementary material available at 10.1186/s12870-023-04421-6.

## Background

Plant growth and development are regulated by a variety of endogenous and exogenous signals. Plant hormones are one of the major endogenous signals that can respond rapidly to environmental stimuli [1]. Strigolactones (SLs), initially found to be secreted from plant roots, act as a germination stimulant for weed seeds and also function in parasitic interactions [2, 3]. SLs are also involved in the development of algae and monocot and dicot plants [4, 5]. In plant growth, SLs regulate stem branching (tillering), leaf senescence, stem secondary wall thickening, photomorphogenesis, and stem elongation, among other [6–9]. Karrikin (KAR)/karrikin-like (KL) is a chemical signal discovered in wildfire smoke, that can effectively stimulate seed germination and seedling growth in *Arabidopsis*. KAR/KL activity depends on the F-box protein MORE AXILLARY BRANCHING (MAX2) in the Skp-Cullin-F-box (SCF) complex [10, 11]. The SCF^MAX2^ complex is involved in regulation of both the SL and KAR signaling pathways, and SL and KAR compounds exhibit partially similar molecular structures and share common biological functions [12]. The receptors for SL and KAR are α/β-hydrolases DWARF14 (D14) and KARRIKIN INSENSITIVE2 (KAI2), respectively [13]. Studies of *Arabidopsis d14* and *kai2* mutants have shown that MAX2 mediates the development processes regulated by SL and KAR/KL [13]. SUPPRESSOR OF MAX2 1 (SMAX1)-LIKE (SMXL) is a candidate target protein of the SCF^MAX2^ complex, which may act downstream of MAX2 and respond to KAR and SL signals [14].

A homolog of *SMAX1*, named *D53*, was identified in a SL-insensitive rice (*Oryza sativa*) mutant and acts as a negative regulator of tillering [15]. The rice D53 homologs, SMAX1, and SMXL6, 7, and 8, are negative regulators of stem branching in the SL signaling pathway, inhibiting the transmission of KAR and SL signals [14, 16]. Exogenous application of the SL analog GR24 leads to the ubiquitination and degradation of SMXL6, 7, and 8. A complex consisting of SMXL, MAX2, and TOPLESS-RELATED PROTEIN2 (TPR2) interacts with D14 in response to GR24 [17]. Treatment of *Arabidopsis* seedlings with GR24 promotes the degradation of SMAX1 and SMXL2 through the D14-SCF^MAX2^ signaling pathway, resulting in MAX2-dependent degradation of different members of the SMXL protein family [18]. The 26S proteasome specifically recognizes the SMXL protein and then is degrades it, thereby preventing the inhibitory effect of downstream transcription factor BRANCHED1 (BRC1) [19]. *AtSMAX1* and *AtSMXL2* regulate the development of roots and root hairs as well as the elongation of hypocotyls in *Arabidopsis*. The complex formed by SMAX1/SMXL2 and MAX2 binds to KAI2, which lead to SMAX1/SMXL2 ubiquitination and degradation of when perceiving KAR signals [20, 21]. Previous research has shown that *SMXL6*, *SMXL7*, and *SMXL8* genes are regulated by KAI2 and are involved in the suppression of abscisic acid (ABA) by inhibiting the *BRC1* gene through the ethylene response factor-associated amphiphilic repression (EAR) motif. This inhibition ultimately promotes branching in plants [22]. KAI2 is not capable of breaking down SMXL6, SMXL7, or SMXL8, but it is able to target SMXL2 [19]. Overall, these studies suggest that D53-like SMXL proteins are part of the SL signaling pathway and play a significant role in plant growth and development, particularly in regulating branching, by responding to KAR and SL signals.

The phosphatidylethanolamine binding proteins, FLOWERING LOCUS T (FT) and TERMINAL FLOWERING L (TFL1), antagonistically regulate flowering transition and the establishment of plant architecture [23]. In *Arabidopsis*, the *FT* gene and the bZIP transcription factor gene *FD* work together to respond to inductive photoperiods and overcome TFL1-FD inhibition of branching. The TFL1-FD complex is identified as a central regulator that restrains reproductive development and endogenous signaling pathways. Recent research has identified the TFL1-FD complex as a hub that plays key roles in inhibiting plant reproductive development [24]. Through large-scale ChIP-seq and functional analysis of downstream genes, TFL1 and FT have been shown to share several important target genes in multiple phytohormone pathways through competition for FD [25, 26]. Components of SL signaling, *SMXL6* and *SMXL8*, may be target genes of the TFL1-FD complex [24]. In particular, TFL1 protein inhibits the target protein SMXL by competing with FT for FD, thus promoting axillary bud branching [24, 27].

Cotton (*Gossypium* sp.) is an important economic crop, and its fruiting branch length and branching pattern have a direct impact on plant architecture, mechanical harvesting, and yield [28–30]. Previous research has identified the involvement of the SL signal in lateral branch development of cotton. For example, ectopic expression of *GhMAX2a* and *GhMAX2b* rescues the dwarf and multi-branch phenotypes of *max2* mutants in *Arabidopsis* [31]. Moreover, protein interaction studies have demonstrated that GhMAX2b interacts with Skp and other proteins to form a SCF-E3 complex, which plays a critical role in lateral branch development [32]. Silencing of *GhMAX2* in cotton results in a dwarf plant phenotype, with slow growth and shortened fruiting internodes and fibers [31]. However, the biological functions and mechanisms of *SMXL* genes in the SL signaling pathway in cotton require further elucidation, and systematic identification and characterization of the *SMXL* gene family in cotton is still lacking.

In this study, we used a homology search to comprehensively explore the evolution of the *SMXL* gene family in 21 green plant species using recently updated genome sequences of Chlorophyta, Bryophyta, and angiosperm groups. We also focused on extensive identification and comparative analysis of *SMXL* gene family members in the genomes of tetraploid (*Gossypium hirsutum* and *Gossypium barbadense*) and diploid (*Gossypium raimondii*, *Gossypium arboreum*, and *Gossypium herbaceum*) cotton genomes, performing comprehensive characterization. Our results lay a foundation for further research on the evolution and function of plant, especially cotton, *SMXL* genes.

## Results

### Identification and evolution of plant *SMXL* gene family members

**Fig. 1:**
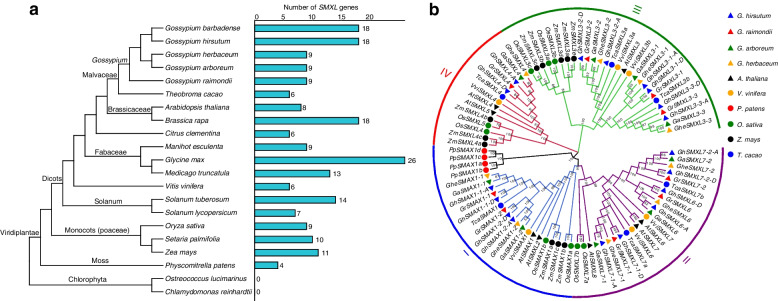
The phylogenetic evolution of plant *SMXL* genes. **a** Phylogenetic tree of *SMXL* genes of 21 representative species from Chlorophyta, Bryophyta, and angiosperm taxa in the plant kingdom; the number of *SMXL* paralogs in each species indicated. **b** Phylogenetic tree of 89 *SMXL* genes in 10 species: *Gh*, *G*. *hirsutum*; *Gr*, *G*. *raymondii*; *Ga*, *G*. *arboretum*; *Ghe*, *G*. *herbaceum*; *At*, *A. thaliana*; *Os*, *O*. *sativa*; *Vvi*, *V*. *vinifera*; *Pp*, *P*. *patens*; *Tca*, *T*. *cacao*; *Zm*, *Z*. *mays*. The phylogenetic tree was reconstructed using the neighbor-joining algorithm in MEGA X software with default parameters provided by MEGA, and the check parameter Bootstrap was repeated 1000 times. I, II, III, and IV represent four clusters

**Fig. 2:**
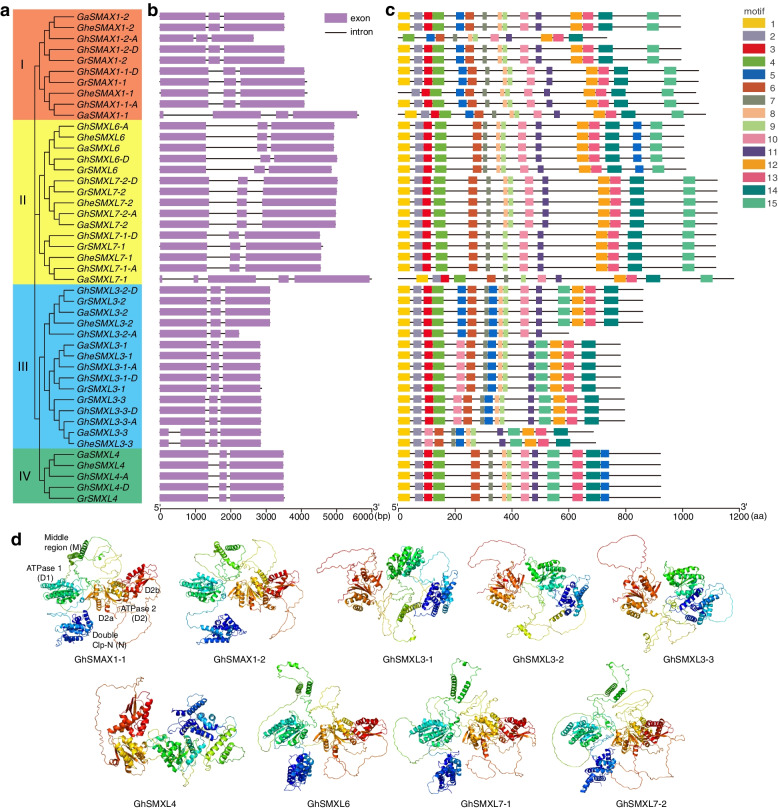
Structure of *SMXL* genes and proteins in *G. hirsutum*, *G. herbaceum*, *G. raymondii*, and *G. arboreum* and 3D analysis of SMXL proteins. **a** Phylogenetic tree of 45 *SMXL* genes in cotton. **b** Intron and exon structures of 45 *SMXL* genes, with purple squares representing exons and lines representing introns. **c** Motif structure of 45 SMXL proteins. **d** 3D structure predictions of nine GhSMXL proteins; motifs are represented by different colors, with N in blue, D1 in light green, M in green, and D2 in red. D2 consists of two subdomains, D2a in light red and D2b in dark red

We identified 210 *SMXL* genes across 21 plant genomes by combining homology comparisons and structural domain searches (Table S1). Phylogenetic analysis of these species indicated that closely related plants were grouped together. Genome-wide analysis revealed a lack of encoded SMXL proteins in green algae, but four potential SMAX1 proteins in moss (Fig. 1a). The information of genome version used in this study was listed in Table S1. Among angiosperms, the number of *SMXL* genes ranged from six in *Vitis vinifera* to 26 in *Glycine max*. Monocot species possessed a similar number of *SMXL* genes: nine in rice (*Oryza sativa*), 10 in millet (*Setaria italica*), and 11 in maize (*Zea mays*). However, the number of *SMXL* genes in dicots varied greatly: for example, six in cocoa (*Theobroma cacao*; Malvaceae), seven in tomato (*Solanum lycopersicum*; Solanaceae), and 14 in potato (*Solanum tuberosum*). Similarly, diploid cotton had nine *SMXL* genes, while tetraploid cotton had 18. In Fabaceae plants, the number of *SMXL* genes also varied greatly, with 26 in soybean (*Glycine max*) and 13 in alfalfa (*Medicago truncatula*). We identified 18, 18, 9, 9, and 9 *SMXL* genes from *G*. *hirsutum*, *G*. *barbadense*, *G*. *herbaceum*, *G*. *raymondii*, and *G*. *arboreum*, respectively. The molecular weights and isoelectric points of 63 SMXL proteins are shown in Table S2. We reconstructed a phylogenetic tree using 89 *SMXL* genes identified in ten species genomes: *Arabidopsis* (8), *Physcomitrella patens* (4), *Vitis vinifera* (6), *O. sativa* (9), *Z*. *mays* (11), *T*. *cacao* (6), *G*. *hirsutum* (18), *G*. *raymondii* (9), *G*. *arboreum* (9), and *G*. *herbaceum* (9) (Fig. 1b). This showed that the 89 *SMXL* genes clustered into four distinct branches (I, II, III, and IV) (Fig. 1b), with clade I containing *SMAX1* and *SMXL2*, while *SMXL6*, *SMXL7*, and *SMXL8* were grouped in clade II. Clade III included only *SMXL3*, and clade IV contained *SMXL4* and *SMXL5*. Moss, an ancient species in evolutionary terms, had only one evolutionary branch of the *SMAX1* gene belonging to clade I. The 11 *SMXL* genes identified in the monocot maize belonged to three distinct evolutionary branches, namely clades I, III, and IV, while the six *SMXL* genes in dicotyledon cocoa and the 45 *SMXL* genes present in cotton species could be classified into four evolutionary branches. Further analysis showed that the *SMXL6*, *7*, and *8* genes in clade II were unique to dicotyledons, with no homologous genes found in monocots. Tetraploid cotton possessed twice as many *SMXL* genes as diploid cotton, which evolutionarily belonged to four branches (Figs. 1b and 2a).

### Cotton *SMXL* gene structure, and amino acid motifs and 3D structure of GhSMXL proteins

We used 45 SMXL proteins from *G*. *hirsutum*, *G*. *herbaceum*, *G*. *raymondii*, and *G*. *arboreum* for phylogenetic analysis. These were clearly clustered into four groups (Fig. 2a), consistent with the above results (Fig. 1b). Gene structure analysis demonstrated a high degree of consistency in gene structure among members of the same subgroup in cotton (Fig. 2b). Most *SMXL* genes contained three exons and two introns, but individual *SMXL* genes had specific structures. For example, in the first subgroup, *GaSMAX1-1* had four exons and three introns, in the second subgroup, *GaSMXL7-1* had five exons and four introns, and in the third subgroup, *GaSMXL3-3* and *GheSMXL3-3*, had four exons and three introns (Fig. 2b). During motif analysis, we select 15 motifs for analysis, which can provide more comprehensive insights into the structural and functional characteristics of SMXL proteins, with a particular emphasis on subgroup’s distinctions. Subsequently, we predicted 15 motif structures between and within *GoSMXL* subgroups in different ploidy cotton species. The available cotton genome includes a tetraploid species, *G*. *hirsutum* (AD_1_), and three diploid species: *G*. *raimondii* (D_5_), *G*. *herbaceum* (A_1_), and *G*. *arboreum* (A_2_), which showed high conservation among and within subgroups of the SMXL protein family (Fig. 2c, Table S3). Despite the deletion of motifs in individual proteins, most members displayed highly conserved motif structures. Motifs 1–4 represented the double Clp-N domain. Motifs 12–15 were located in the SMXL C-terminal region, showing correspondence with the P-loop NTPase domain. All identified EAR motifs were located within motif 12. Clp-N domains and P-loop NTPase were highly conserved in *G*. *hirsutum*, with the exception of *GhSMXL3-2-A*, which displayed a conserved EAR motif (Fig. S1). We further predicted the three-dimensional (3D) structure of the GhSMXL proteins based on the AlphaFold2 program [33] (Fig. 2d). Nine pairs of GhSMXL proteins were classified into four types based on their 3D structures: GhSMAX1-1/GhSMAX1-2, GhSMXL3-1/GhSMXL3-2/GhSMXL3-3, GhSMXL4, and GhSMXL6/GhSMXL7-1/GhSMXL7-2. Additionally, five core domains of GhSMXL protein models were defined according to the naming of Shabek et al. (2018) and Zhou et al. (2013): N-terminal Double Clp-N motif (N), hypothetical ATPase domain 1 (D1), middle region (M), and C-terminal hypothetical ATPase domain 2 (D2), which is further divided into D2a and D2b subdomains [34]. These findings suggest that the higher the amino acid similarity between proteins, the more similar their 3D structures, and possibly the more similar their functions.

### Chromosomal distribution, collinearity, and selection pressure analysis of the cotton *SMXL* gene family

The *SMXL* gene family members were found to be distributed on specific chromosomes in tetraploid cotton species *G*. *hirsutum* (AD_1_) and *G*. *barbadense* (AD_2_) (Fig. S2), consistent with their distribution in AD_1_ and AD_2_. Specifically, *SMXL* genes were found on chromosomes D04, A05, D05, A08, D08, A09, D09, A10, D10, A13, and D13 in both tetraploid species. In *G*. *raimondii* (D_5_), these genes were located on chromosomes 6, 7, 9, 11, and 13, whereas in *G*. *arboreum* (A_2_), they were located on chromosomes 04, 05, 07, 08, 09, 10, and 13. Similarly, in *G*. *herbaceum* (A_1_), these genes were located on chromosomes 5, 7, 8, 9, 10, and 13. Notably, chromosome A05 in both tetraploid species had the highest number of *SMXL* gene family members, with four genes located on each chromosome. Diploid species, represented by *G*. *arboreum*, *G*. *herbaceum*, and *G*. *raimondii*, had three *SMXL* genes located on chromosomes 5 and 13. Notably, *GheSMXL3-1* (*GheUnG00160*) in *G*. *herbaceum* was not located on a specific chromosome, but on unassembled Contig1004. In addition, most genes except *GhSMXL7-2* were distributed at the ends of chromosomes. However, one homeologous gene pair, *GhSMXL3-1-A*/*D*, was located on distinct A and D chromosomes. *GhSMXL3-1-A* locates at the end of chromosome 5, while *GhSMXL3-1-D* locates at the end of chromosome 4. The same phenomenon was observed for the *G*. *barbadense GbSMXL3-1-A/D* gene pair. These findings suggest that the chromosome segment of these genes may have undergone translocation during the evolution of tetraploid cotton.

**Fig. 3:**
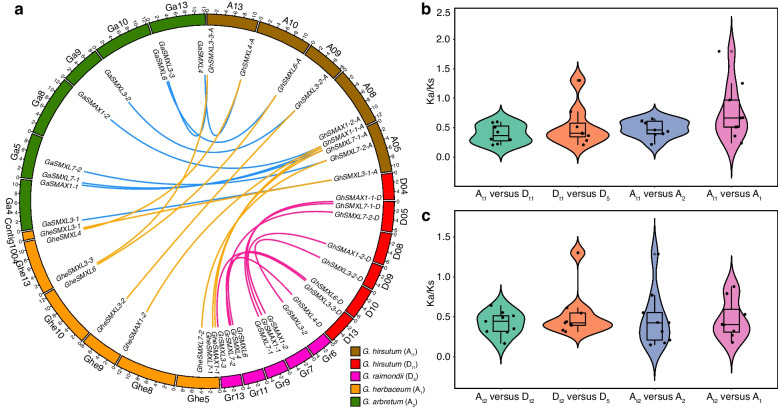
Analyses of collinearity and evolution between diploid and tetraploid cottons. **a** Colinear analysis between *G*. *hirsutum* (AD_1_) and *G*. *raimondii* (D_5_), *G*. *arboreum* (A_2_), and *G*. *herbaceum* (A_1_), respectively. The outermost ring indicates the different chromosomes, indicated by numbers. Different colors indicate homologous gene pairs between tetraploid and diploid cottons. *G*. *herbaceum CheSMXL3-1* gene is mapped in Contig1004; blue lines connect *G*. *hirsutum* and *G*. *arboreum* gene pairs, yellow lines connect *G*. *hirsutum* and *G*. *herbaceum* gene pairs, and red lines connect D_t_ and D_5_ gene pairs. **b** *K*a/*K*s values of *SMXL* homologous gene pairs between upland and diploid cottons. **c** *K*a/*K*s values for *SMXL* homologous gene pairs between island and diploid cottons

The D_t_ genome of allotetraploid cotton originated from *G*. *raimondii*, while the A_t_ genome is thought to be derived from the common ancestor of *G*. *arboreum* and *G*. *herbaceum* [35]. To investigate the expansion and contraction of the *SMXL* gene family during evolution, we identified the collinear gene pairs of diploid cotton in tetraploid cotton. We further analyzed information on 63 *SMXL* gene pairs discovered in tetraploid cotton (AD_1_ and AD_2_) and diploid cotton (D_5_, A_1_, and A_2_) (Table S4). Using MCScanX software [36], we subsequently reconstructed the collinear relationships between tetraploid and diploid cottons (Fig. 3a) and found that nine *SMXL* orthologs of upland (tetraploid) cotton were present in the genomes of diploid cotton (A_1_ and A_2_). Similarly, analysis of collinear relationships between *SMXL* genes of island (tetraploid) and diploid cotton revealed that there were also nine homologs of *G*. *barbadense SMXL* genes in diploid cotton genomes (Fig. S3). These results suggest that the *SMXL* genes in tetraploid and diploid cottons have been highly conserved during evolution.

We further identified orthologous genes between tetraploid and diploid cottons, and then analyzed selection pressures on the *SMXL* gene family in cotton by calculating the ratio of non-synonymous to synonymous substitution rates (*K*a/*K*s) of orthologous gene pairs. The *K*a/*K*s values of all upland cotton and diploid cotton colinear gene pairs were between 0.2 and 0.7 (Fig. 3b), except for *GhSMXL3-3-D* and *GrSMXL3-3*, which was 1.30336 (Table S5); in addition, the *K*a/*K*s values of all colinear gene pairs of island cotton and diploid cotton were less than 1 (Fig. 3c), indicating that most *SMXL* gene family members were mainly subjected to purifying selection during evolution. Duplication type analysis revealed that segmental duplications were the main mechanisms for producing the expansion of the *SMXL* gene family in cotton (Table S5).

### Expression patterns of *GhSMXL* genes in *G. hirsutum*

**Fig. 4:**
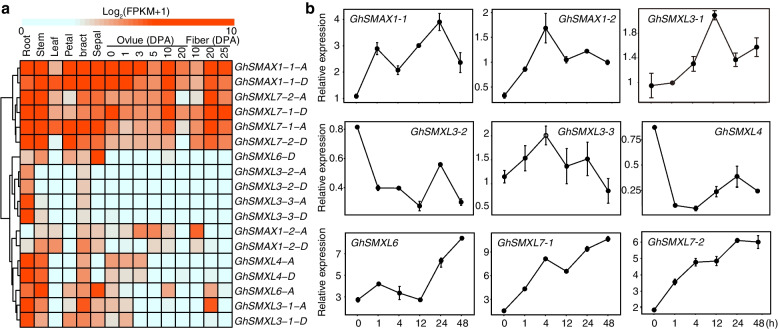
Expression characteristics of *GhSMXL* genes. **a** Expression heatmap of *GhSMXL* genes in different tissues of *G*. *hirsutum*. **b** Expression levels of *GhSMXL* genes after GR24 treatment; upland cotton seedlings cultured for 16 days were treated with 10 μM GR24 for 0 h (control), 1, 4, 12, 24, and 48 h. Error bars indicate means ± SD (*n* = 3)

To investigate the expression patterns and potential biological roles of *GhSMXL* genes in upland cotton, we performed comprehensive tissue-specific expression analysis using RNA-seq data (Fig. 4a). The expression heatmap revealed three distinct classes among the 18 *GhSMXL* genes, according to their expression patterns across 15 different tissues. The first group of genes (*GhSMAX1-1-A*/*D*, *GhSMXL7-1-A*/*D*, and *GhSMXL7-2-A*/*D*) exhibited high expression levels in six different tissues and organs (root, stem, leaf, petal, receptacle, and sepal), as well as in fiber-bearing ovules at four time points [0, 1, 3, and 5 d post-anthesis (DPA)], ovules of 10 and 20 DPA, and fibers at three time points (10, 20, and 25 DPA). The second group of genes, consisting of *GhSMXL3-2-A*/*D* and *GhSMXL3-3-A*/*D*, was expressed at high levels primarily in roots, with minimal expression observed in other tissues. By contrast, *GhSMXL6-D* was detected in multiple tissues, including roots, stems, petals, receptacles, and sepals, with the highest expression level observed in sepals. The third class of genes contained seven genes, consisting of *GhSMXL1-2-A*/*D*, *GhSMXL3-1-A*/*D*, *GhSMXL4-A*/*D*, and *GhSMXL6-A*, most of which were mainly expressed in roots and stems, except for *GhSMXL1-2-A*/*D*. *GhSMAX1-2-A* showed the highest expression level in fibers at 10 DPA, followed by high expression in 3- and 5-day-old ovules, and moderate expression in stems, leaves, and receptacles, with low or no expression in other tissues. Additionally, *GhSMXL6-A* was highly expressed in sepals, and *GhSMXL3-1-A* was highly expressed in receptacles and fibers at 20 DPA. In general, the 18 *GhSMXL* genes displayed different expression patterns in various tissues, organs, and fiber developmental stages. Some homeologous gene pairs showed distinct tissue-specific expression differences, suggesting the potential functional diversification of the *GhSMXL* gene family.

Subsequently, we performed qRT-PCR experiments to further verify the reliability of the transcriptome data. We analyzed nine *GhSMXL* homeologous gene pairs (without distinguishing between gene pairs on the A and D donor). We used roots, stems, leaves, flowers, shoot apical meristems (SAMs), fiber-bearing ovules ( -3, 0, 3, and 5 DPA), and fibers (8, 12, 20, and 30 DPA) for expression validation. The expression levels of most genes were consistent with the RNA-seq data (Fig. S4). *GhSMAX1-1* was highly expressed in leaves and its expression level in 20 DPA fibers showed a rapid increase, corresponding to RNA-seq data. *GhSMXL3-1* had relatively high expression levels in stems, but its expression level in roots was the highest in the RNA-seq data, with relatively low expression in other tissues. *GhSMXL3-2* had relatively high expression in roots, with the lowest expression at the beginning of fiber development, consistent with the RNA-seq data. *GhSMXL3-3* had relatively high expression in roots, stems, and leaves, and low expression in ovules and at different periods of fiber development, whereas the RNA-seq data showed high expression only in roots. *GhSMXL4* was highly expressed in roots and SAM, with low expression in ovules and during fiber development, consistent with RNA-seq data. The expression patterns of *GhSMXL6*, *GhSMXL7-1*, and *GhSMXL7-2* were similar, with high expression only in stems and relatively low expression in other tissues and at different developmental stages. In short, the expression characteristics of *GhSMXL* genes revealed by RNA-seq and qRT-PCR assays suggested that *GhSMXL* genes regulate multiple aspects of cotton growth and development and may show functional differentiation.

We analyzed the expression of *GhSMXL* genes at different time points using qRT-PCR after treatment with the SL analogue GR24 for 48 h. Nine *GhSMXL* genes showed substantial responses to GR24 treatment and exhibited different expression patterns (Fig. 4b). In the first subgroup, the expression levels of *GhSMAX1-1* and *GhSMAX1-2* genes increased after GR24 treatment. Among them, *GhSMAX1-1* gene expression decreased at 4 h after rising and peaked at 24 h, then declined slowly. However, *GhSMAX1-2* expression first rose and reached its peak at 4 h, then declined slowly. In the second subgroup, the expression levels of *GhSMXL6*, *GhSMXL7-1*, and *GhSMXL7-2* gradually increased within 48 h after GR24 treatment (Fig. 4b), reaching their peak of 6–8 times that of the control at 48 h. The third subgroup displayed differential expression patterns. *GhSMXL3-1* expression gradually increased at first, peaked at 12 h, but remained high. *GhSMXL3-2* expression decreased sharply after GR24 treatment and showed a decreasing trend over time. *GhSMXL3-3* expression rose at first, peaking at 4 h, then declined, increasing slightly at 24 h, and reached its lowest level at 48 h, showing a decline in general. However, the expression level of *GhSMXL4* in the fourth subgroup was similar to that of *GhSMXL3-2*, showing a sharp decline at first after GR24 treatment, then a slow rise, and finally a decline. These findings suggested that *GhSMXL* genes respond to SL signals. We speculate that *GhSMXL6*, *GhSMXL7-1*, and *GhSMXL7-2* genes are regulated by SL phytohormone signals and may play important roles in the growth and development and phytohormone pathways of cotton.

### Co-expression network of SMXL6/7–1/7–2 proteins in *G. hirsutum *and *G. arboreum*

**Fig. 5:**
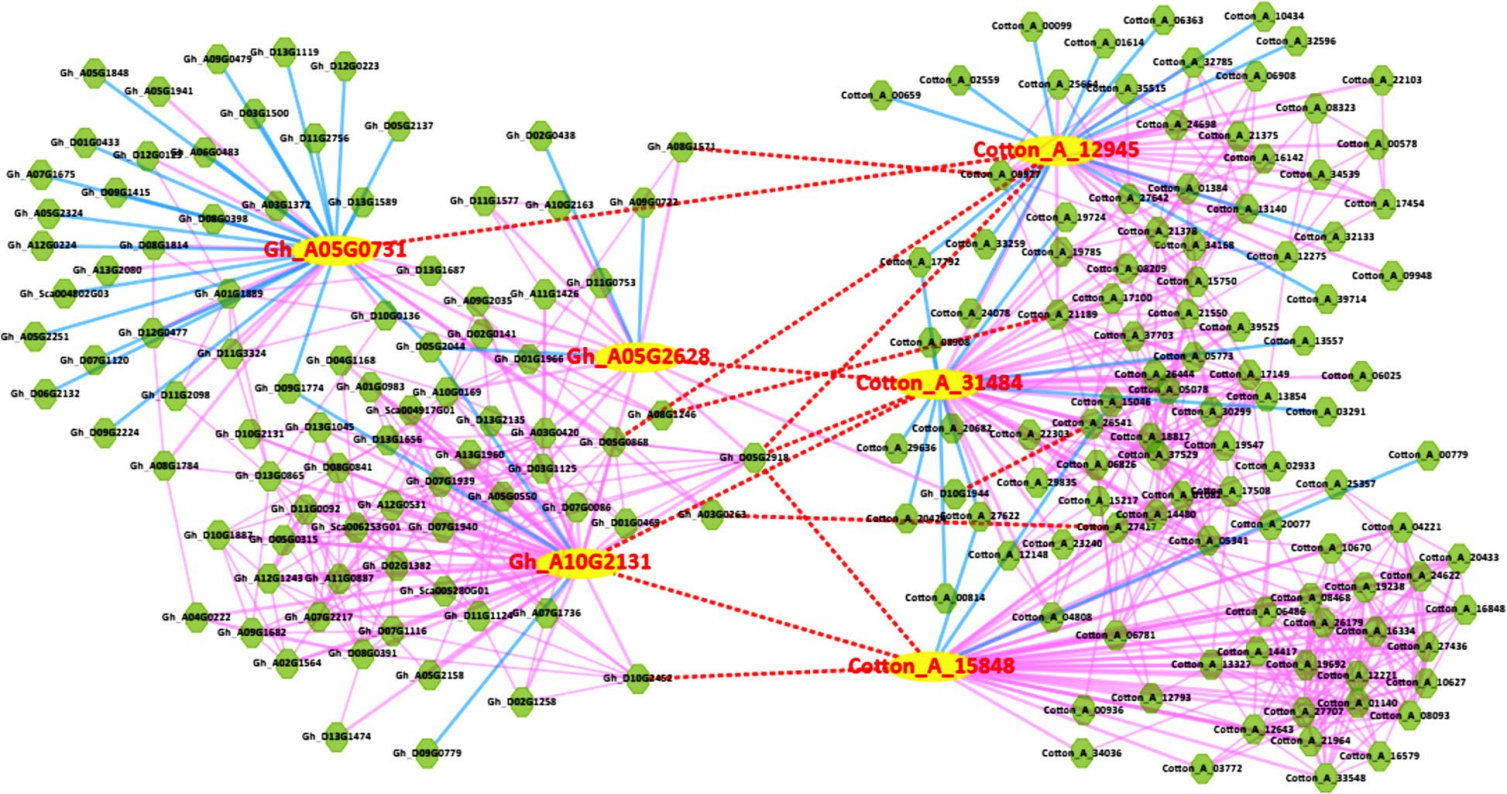
Comparative analysis of the co-expression networks of SL signaling pathway target proteins SMXL6/7–1/7–2 in *G*. *hirsutum* and *G*. *arboreum*. Proteins encoded by homologous gene pairs in the two species are connected by red dashed lines. Yellow indicates key proteins. The key proteins in *G*. *hirsutum* are Gh_A10G2131 (GhSMXL6), Gh_A05G0731 (GhSMXL7-1), and Gh_A05G2628 (GhSMXL7-2), while the key proteins in *G*. *arboreum* are Cotton_A_15848 (GaSMXL6), Cotton_A_12945 (GaSMXL7-1), and Cotton_A_31484 (GaSMXL7-2), which are noted in red font. Green indicates target proteins, which interact with SMXL6/7–1/7–2. Solid pink lines connect up-regulated proteins, and solid blue lines connect down-regulated proteins

In previous studies, *Arabidopsis* SMXL6/7/8 and rice D53 proteins have been shown to promote branching in the D14-MAX2 signaling pathway activated by SL through ubiquitination-mediated protein degradation [14, 17]. To investigate the potential biological functions of *SMXL6*, *7*, and *8* in cotton, we further explored the co-expression networks of *GhSMXL6*, *GhSMXL7-1*, and *GhSMXL7-2* with their homologs *GaSMXL6*, *GaSMXL7-1*, and *GaSMXL7-2* in *G*. *hirsutum* and *G*. *arboreum* (Fig. 5). The SMXL6, SMXL7-1, and SMXL7-2 proteins of *G*. *hirsutum* and *G*. *arboreum* and proteins encoded by their co-expressed genes were annotated and functionally classified (Table S6). GhSMXL6/7–1/7–2 and GaSMXL6/7–1/7–2 proteins contain two Clp-N motifs and a P-loop structure of hydrolases (Table S6). Functional predictions indicated that the proteins interacting with GhSMXL6/7–1/7–2 and GaSMXL6/7–1/7–2 are mainly involved in bud development and branching, including WUSCHEL (WUS) [37], BELL1 (BEL1) [38], and myb domain protein 5 (MYB5) [39]; transcription factors involved in the regulation of plant growth and development, such as basic leucine zipper (bZIP) [40], GRAS [41], and AP2/ERF [42]; proteins related to phytohormone signaling pathways such as gibberellin 2-beta-dioxygenase 2 (GA2OX2) [43] and GA-stimulated transcript 1 (GAST1) [44], involved in the gibberellin pathway; proteins that respond to red/far-red light and anthocyanin formation, such as cryptochrome circadian regulator 1 (CRY1) [45] and cytochrome P450 (P450) [46]; proteins involved in metabolic biosynthesis pathways, such as trehalose-6-phosphate synthase (TPS) [47], hexokinase 2 (HXK2) [48], and dehydration-responsive element-binding (DREB) [49]. These results suggest that the target proteins of GhSMXL6/7–1/7–2 are highly expressed in buds and floral organs and participate in biological processes related to branching development, phytohormone signaling, light response, and metabolic biosynthesis pathways, which provides a basis for further investigations into the functional mechanism of *SMXL6/7–1/7–2* genes.

### Silencing of *GhSMXL* genes inhibits cotton growth

**Fig. 6:**
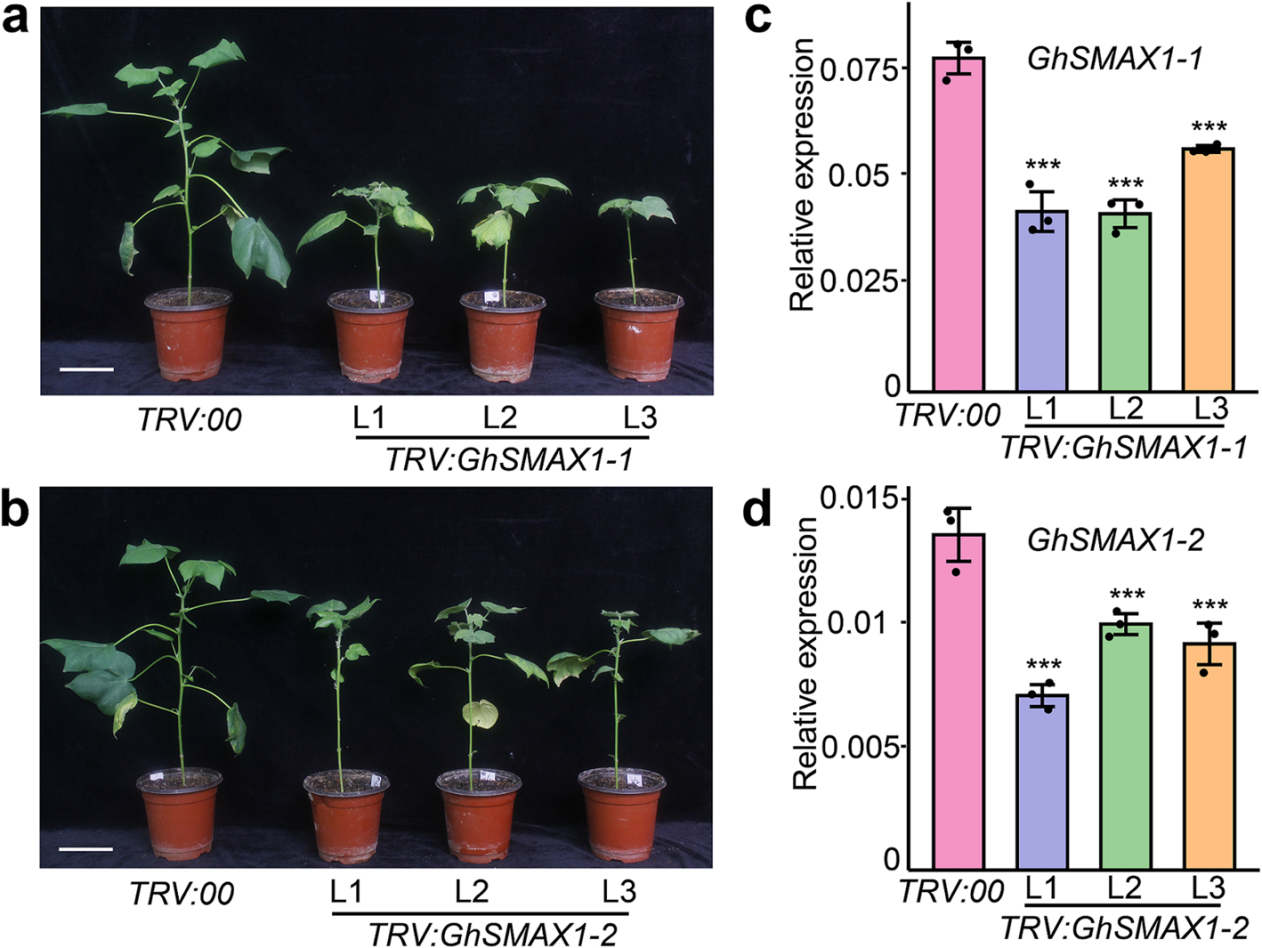
VIGS assays of *GhSMAX1-1* and *GhSMAX1-2* genes in cotton. **a**, **b** Phenotypes resulting from silencing of the *GhSMAX1-1*/*2* genes in upland cotton; bars, 10 cm. Relative expression of *GhSMAX1-1* (**c**) and *GhSMAX1-2* (**d**) among control and VIGS plants using qRT-PCR. Values are means ± SD (*n* = 3). Asterisks indicate significant differences between control TRV:00 and TRV: *GhSMAX1-1*/*2* silencing lines (Student’s *t* test, “***” represents *P* < 0.001)

To investigate the roles of *GhSMXL* genes in regulating cotton growth and development, we used the virus-induced gene silencing (VIGS) method to silence *GhSMAX1-1* and *GhSMAX1-2* genes. After silencing of two *GhSMXL* genes, phenotypic observations showed that growth of the apical meristem was slow or stagnant, development of axillary buds was inhibited, and plant height was dwarfed compared with the control (Fig. 6, Fig. S5). The TRV: *SMAX1-1* plants showed an obvious dwarfing phenotype, and growth of the main stem was inhibited (Fig. 6a). The TRV: *SMAX1-2* plants also showed dwarfing and delayed growth (Fig. 6b). These results suggest that *GhSMXL* genes play crucial roles in regulating cotton growth and development. Silencing of the *GhSMAX1-1* or *GhSMAX1-2* genes had a substantial impact on the growth of the apical meristem and the elongation of the stem, ultimately causing dwarfism in cotton plants. qRT-PCR assays revealed that the expression levels of *GhSMAX1-1*/*2* were significantly reduced in silenced plants compared with the control group (Fig. 6c and d). Taken together, these results suggest that *GhSMAX1-1*/*2* genes play important roles in promoting stem elongation and axillary bud development in cotton.

## Discussion

### Evolution of the *SMXL* gene family in plants

In recent years, owing to the development of genome sequencing, comprehensive genome-wide analyses of *SMXL* genes haves been conducted in several species, with 8, 10, 31, and 12 *SMXL* gene family members identified in *Arabidopsis*, apple, soybean and poplar, respectively [50–53]. Moturu et al. (2018) dissected the expansion of the *SMXL* gene family in 58 plant genomes and found that the oldest *SMAX1* gene underwent three duplications during the evolutionary process from bryophytes to land plants, resulting in the development of new genes or functional differentiation. Moturu et al. (2018) revealed that the expansion of *SMXL* genes likely involved 128 duplications and 276–284 loss events [50]. Whole-genome duplication (WGD) and whole-genome triplication (WGT) mechanisms may have played a critical role in this process. It is postulated that the *SMXL* genes first appeared in liverworts. Previous studies have shown that the *SMAX1* gene family emerged in a terrestrial plant (moss), and it is speculated that the *SMXL* gene family originated from Bryophyta [5]. The results of molecular clock tests indicate that the members of the four branches of the *SMXL* gene family have undergone functional differentiation or neo-functionalization, indicated by their different evolutionary rates [50]. Using fine-assembled plant genomes and diverse plant species, this study further introduced diploid and tetraploid cotton genomes, identifying 210 *SMXL* genes in 21 representative species from Chlorophyta, Bryophyta and angiosperms. Aquatic Chlorophyta do not contain any *SMXL* genes, and only four *SMAX1* genes were identified in bryophytes. Bryophytes are considered to be the earliest plants to differentiate into stems and leaves, suggesting that the *SMAX1* genes may play an important role in plant morphology formation and adaptation to environmental changes. Our results suggest that gene duplication is the primary driver of *SMXL* gene evolution in cotton, as the number of *SMXL* gene families in tetraploid cotton is twice that of diploid cotton. Phylogenetic analysis showed that plant *SMXL* genes can be classified into four branches (I, II, III, and IV) (Fig. 1b, Fig. 2a), with only one *SMAX1* branch identified in bryophytes, consistent with previous research [50]. Moreover, we found that *SMXL6*, *SMXL7*, and *SMXL8* in the second subgroup of angiosperms are unique to dicotyledonous plants, suggesting that cotton *SMXL6*, *SMXL7-1*, and *SMXL7-2* may play vital roles in species differentiation.

### Structural characteristics and evolution of the *SMXL* gene family in cotton

Since the successful completion of the first *G*. *raimondii* genome sequencing and assembly in 2012 [36], 41 cotton genomes have been assembled and published in the past decade [54]. These genomic resources have significantly advanced cotton functional genomics research. The D subgenome of *G*. *hirsutum* originates from *G*. *raimondii* D_5_, while the A subgenome comes from the common ancestor A_0_ genome of *G*. *herbaceum* and *G*. *arboreum*. *G*. *arboreum* did not originate from *G*. *herbaceum* but was independently domesticated from an unknown A_0_ genome [35]. In this study, we identified 63 *SMXL* genes from the genomes of two tetraploid and three diploid cotton species. The presence of nine *SMXL* genes in diploid cotton and 18 *SMXL* genes in tetraploid cotton indicates that all *SMXL* genes in tetraploid cotton were generated by genome duplication. The distribution of *SMXL* genes on chromosomes is consistent between *G*. *hirsutum* and *G*. *barbadense* (Fig. S2). Notably, *SMXL3-1* in the A and D subgenomes of tetraploid cotton is found at the ends of chromosomes 5 and 4, respectively. We hypothesize that the chromosome segment containing the *SMXL3-1* gene underwent translocation during the process of cotton genome duplication. No tandem duplication was found in the cotton *SMXL* gene family, with most family members arising from segmental duplication. This suggests that segmental duplication events were the primary driver of expansion in the *SMXL* gene family in cotton. All diploid cotton species have nine *SMXL* genes that correspond to those in tetraploid cotton, suggesting that the *SMXL* gene family is highly conserved in cotton. Amino acid changes caused by nonsynonymous substitutions can result in alterations in protein conformation and function [55]. The majority of *SMXL* genes have a *K*a/*K*s value of less than 1, indicating that members of the *SMXL* gene family were under selective pressure for adaptation to environmental changes during cotton tetraploidization. Most *SMXL* genes have been retained by purifying selection, suggesting their crucial roles in species differentiation.

The evolutionary characteristics and functional differentiation of homologous *SMXL* genes can be attributed to the presence of consistent and differential functional motifs in their conserved structure and peptide sequences [56]. Within the same subgroup, the structure of genes and protein motifs are similar, with a few exceptions (Fig. 2b and c). Apart from *GaSMAX1-1*, *GaSMXL7-1*, *GaSMXL3-3*, and *GheSMXL3-3*, all other *SMXL* genes possess three exons and two introns. There are differences in the number of motifs and amino acids in different subgroups, but the Clp-N domains, P-loop NTPase, and EAR-like motifs of their amino acid sequences are relatively conserved (Fig. S1), which may reflect different biological functions between each subgroup. 3-D structures of SMXL proteins in *G*. *hirsutum* have revealed that proteins of the same subgroup exhibit a high degree of structural similarity. Moreover, the N, D1, M, and D2 domains of these proteins are likely to play a crucial role in performing specific functions [57]. Previous studies demonstrated that the *Arabidopsis* SMXL7_N_ domain contains a nuclear localization signal. The D2 domain of SMAX1 serves as a target for degradation induced by KAR and SL [34, 58]. Yeast two-hybrid experiments with SMAX1 and SMXL7 indicate that the SMXL protein interacts with KAI2 and D14 via the D1M domain, while the N domain is unnecessary [34]. A conserved Arg-Gly-Lys-Thr (RGKT) motif contained in the D2a domain is crucial for MAX2-mediated D53/SMXL7 protein degradation. SMXL3, SMXL4, and SMXL5 proteins cannot be degraded because they lack a RGKT motif [14]. Moreover, SMAX1 and SMXL2 lose their SCF^MAX2^-induced degradation activity after RGKT motif deletion [34]. We found that protein structures in the same subgroup are highly similar, and the main domain (N, D1, M, and D2) of these proteins may play a key role in their specific functions. This study therefore provides valuable insights into the evolution of the plant *SMXL* gene family and our understanding of the structural characteristics of this family, which will facilitate further exploration of the biological functions of SMXL proteins.

### Cotton *SMXL* genes regulate various aspects of growth and development

Previous research revealed that members of sub-group I (AtSMAX1/AtSMXL2) play a critical role in the KAR signaling pathway and directly participate in the regulation of seed germination and leaf development in *Arabidopsis* [14, 59]. These proteins, together with SMXL2, also function downstream in the KAI signaling pathway to regulate root and root hair development in conjunction with SMXL2 [60]. They also promote hypocotyl elongation, resulting in crosstalk between the D14-SMAX1 signal and targeted degradation of SMAX1 protein under GR24 treatment and osmotic stress [61]. By contrast, members of sub-group II (AtSMXL6, 7, 8, and rice D53) encode key inhibitors of the SL signaling pathway. Upon activation of the SL signaling pathway, SMXL6/7/8 proteins are degraded by proteasomes, leading to the inhibition of branching in *Arabidopsis* and tillering in rice [14, 17, 57]. Members of sub-groups III and IV (AtSMXL3, AtSMXL4, and AtSMXL5) exhibit different characteristics from other members of the SMXL family; they are not involved in the SL or KAR signaling pathways and do not rely on MAX2-mediated protein degradation [18]. Recent research shows that *SMXL* genes have a wide range of functions in plant growth, development, and stress responses [62]. However, the molecular mechanisms underlying phytohormone signaling and abiotic stress responses remain poorly understood. Analysis of the upstream 2000-bp sequence of the *GhSMXL* gene transcription start site revealed the presence of various cis-acting elements associated with light, growth and development, phytohormones, and stress (Fig. S6). This suggests that *GhSMXL* genes may execute functional diversity. *GhSMXL* genes display diverse patterns of expression across various tissues, organs, and stages of fiber development, with homologous genes exhibiting similar expression characteristics but individual genes showing differential expression. GR24 treatment of cotton seedlings produces a stable increase in the expression levels of *SMXL6*/*7–1*/*7–2* genes, and rising trends in the expression levels of *GhSMAX1-1* and *GhSMAX1-2* genes, albeit with fluctuating expression levels. Co-expression network analysis identified proteins that may interact to perform a specific function; for example, the anti-florigen protein TFL1, Cryptochrome Circadian Regulator 1 (CRY1), cytochrome enzyme P450, meristem-associated WUSCHEL protein, gibberellin biosynthesis-related gibberellin 2-beta-dioxygenase 2 (GA2OX2), and GA-stimulated transcript 1 (GAST1) proteins, all of which are involved in phytohormonal signaling pathways. According to the expression characteristics of *GhSMXL* genes in different tissues and after GR24 treatment, we speculate that cotton *SMXL6*/*7–1*/*7–2* genes play critical roles in the SL signaling pathway with some functional redundancy. These results imply that the members of the cotton *SMXL* gene family respond to SL signals and participate in plant growth and development.

### GhSMAX1-1/2 play an important role in the regulation of cotton growth

The D14/KAI-SMXL complex interacts with SCF^MAX2^, resulting in degradation of SMXL proteins [14]. D14 and KAI receptors need F-box protein MAX2 to perceive signaling molecules. *Arabidopsis* SMAX1 and its homologous protein rice D53 serve as downstream targets of MAX2. The *Arabidopsis max2* mutant has a dwarf stature and increased lateral branching phenotype, while *SMXL6*, *7*, and *8* can restore *max2* to the wild-type phenotype [14]. Thus, SMXL6, 7, and 8, as target proteins of MAX2, act as suppressors of SL signals, functioning in the regulation of plant height and branching. The *Arabidopsis smax1 smxl2* mutant has a very short hypocotyl. Genetic evidence shows that degradation of SMXL6, 7, and 8 proteins does not affect hypocotyl growth, while interactions between SMAX1, SMXL2, and D14 promote hypocotyl elongation in response to GR24 in *Arabidopsis* [61]. We found that downregulation of *GhSMAX1-1* and *GhSMAX1-2* genes through VIGS inhibits stem elongation and axillary bud development, resulting in a significant decline in cotton plant height. These results further confirm that *SMAX1* homologs play critical roles in regulating hypocotyl elongation to change plant architecture by affecting plant height.

## Conclusions

In this study, we identified 210 *SMXL* genes from 21 plant species and divided these into four different phylogenetic clades with functional diversities. Homologs of *SMAX1* have been well conserved during the evolution of terrestrial plants. Among them, 63 *SMXL* genes identified from five *Gossypium* species were clustered into four clades, and all cotton SMXL proteins contained conserved Clp-N domains, P-loop NTPases, and EAR motifs. The *GhSMXL* gene is highly expressed in root and stem tissues, and the expression level of *GhSMXL6*/*7–1*/*7–2* is considerably responsive to GR24 treatment. Protein network analysis showed that the target proteins of SMXL6, SMXL7-1, and SMXL7-2 in *G. hirsutum* and *G. arboreum* are involved in the development of shoots and floral organs. Silencing of *GhSMAX1-1* and *GhSMAX1-2* genes resulted in plant dwarfism and inhibition of axillary bud development. The present study suggests functional diversities of the plant *SMXL* gene family and the important roles of GhSMXL in the SL signaling pathway. Rapid technological progress should enable us to dissect the functional specificity of plant SMXL proteins to understand their importance in cotton development.

## Methods

### Plant materials

In summer of 2019, *G. hirsutum* L. cv. ‘XLZ 33’ were field-grown under natural conditions at Shihezi University's experimental farm in Shihezi City, Xinjiang, China (44°20′ N, 86°0′ E), as previously described [63]. We have obtained the permission to collect plant material *G. hirsutum*. Samples of roots, stems, true leaves, and SAM at 40 days post-planting and flowers at -3, 0, 3, and 5 days post-anthesis (DPA), as well as fibers at 8, 12, 20, and 30 DPA, were collected, respectively. Seeds of *G*. *hirsutum* L. standard line TM-1 were grown in Petri dishes with nutrient soil and vermiculite mixture (1:1). Petri dishes were placed in a growth chamber (16-h light/8-h dark, 200 µmol m^–2^ s^–1^). Plants growing for 14 days with 2 days of 50 ml Hoagland nutrient solution irrigation were treated with rac-GR24 (10 μM) (purchased from Solarbio) by wiping the cotton leaves [64]. Leaves from three consistent, young seedlings were collected as three biological replicates at 0, 1, 4, 12, 24, and 48 h after GR24 treatment. All tissues samples were frozen immediately in liquid nitrogen and stored at -80 ℃ for RNA extraction.

### Identification of *SMXL* gene family members in 21 plant species

Genome sequences of five cotton species, *G*. *hirsutum*, *G. barbadense*, *G. raimondii*, *G. arboreum*, and *G. herbaceum*, were obtained from the cotton genome database (table S7). Genome sequences of other Viridiplantae—*P*. *patens*, *V*. *vinifera*, *O*. *sativa*, *Z*. *mays*, *T*. *cacao*, *O*. *lucimarinus*, *C*. *reinhardtii*, *S*. *italica*, *S*. *lycopersicum*, *S*. *tuberosum*, *M*. *esculenta*, *M*. *truncatula*, *Brassica rapa*, *Cucumis sativus*, *Glycine max*, and *C*. *clementina*—were downloaded from the Phytozome database (https://phytozome.jgi.doe.gov/pz/portal.html). The protein sequences of SMXLs in *A*. *thaliana* were obtained from the TAIR website (https://www.Arabidopsis.org) and used as probes for homology searches with the BLASTP program (*E*-value < 1 × 10^−5^). Protein sequences identified were submitted to the PfamScan program (https://pfam.xfam.org/) to obtain candidate SMXL proteins using domain analysis. *SMXL* homologs in cotton were classified and named according to their similarity to *A*. *thaliana* and the structural characteristics of genes. Molecular weight and isoelectric point of proteins were estimated using the ExPASy protein analysis tool (https://web.expasy.org/), and subcellular localization was predicted using the ProtComp 9.0 program (http://linux1.softberry.com/berry.phtml).

### Phylogenetic tree reconstruction, gene structure, and protein motif distribution

Divergence times of the above 21 plant species on a scale of MYA (million years ago) with confidence intervals were estimated using the TIMETREE website (http://www.timetree.org/). A Newick file was generated, and a species phylogenetic tree was reconstructed using MEGA11 software [65]. Members of the *SMXL* gene family were identified and clustered using ClustalW software for multiple sequence alignment of protein sequences with default parameters [66]. A phylogenetic tree of the *SMXL* genes was reconstructed using the neighbor-joining (NJ) method in MEGA11 software [65] to analyze their evolutionary relationships. The positions of introns and exons in the *SMXL* genes in cotton were extracted from the gff file of the cotton database. Multiple sequence alignment was performed using MUSCLE software (https://www.ebi.ac.uk/Tools/msa/muscle/), and conserved motifs in the protein sequences were identified using the online MEME website (http://meme-suite.org/meme) with 15 motifs and default settings. Gene structure diagrams were drawn using TBtools software [67].

### Analysis of *cis*-acting elements and prediction of 3D protein structure

Promoter sequences were obtained by considering the 2000 bp upstream of the start codon. Plant Cis-acting Regulatory Element (Plant CARE) software [68] was used to search for *cis*-acting regulatory elements, and the results were visualized using the R package pheatmap. Protein structure prediction was performed using the AlphaFold2 program [33] based on known amino acid sequences of the protein family; an initial model was generated, followed by outputting the protein 3D structure in the PyMOL software (https://pymol.org/2/).

### Chromosome localization

Chromosome positions of *SMXL* genes were determined by identifying their start and end positions from the cotton genome database gff3 file. A chromosome location map was designed using MapGene2C online software (http://mg2c.iask.in/mg2c_v2.1/) [69].

### Comparative evolutionary analysis

Collinearity analysis was conducted to predict homologous genes of *SMXL* between species using MCScanX [70] with default parameters. Orthologous genes between diploid and tetraploid cotton were identified using BLASTP alignment with E-value < 1 × 10 − 5. Duplication types of the cotton *SMXL* gene family were analyzed using the downstream analysis program of MCScanX software, duplicate_gene_classifier [70]. Circos software [71] was used to visualize collinearity relationships of homologous genes. Homologous genes were aligned using the Muscle method in paraAT2.0 software [72]. The non-synonymous substitution rate (*K*a), synonymous substitution rate (*K*s), and *K*a/*K*s ratio were calculated using *K*a*K*s_Calculator2.0 [73] to evaluate the selective pressures on *SMXL* genes during the evolutionary process. A Ka/Ks ratio > 1, < 1, or = 1 indicates positive, negative, or neutral evolution, respectively [74].

### Expression profiles of *GhSMXL* genes

RNA-seq data of the different tissues of *G*. *hirsutum* TM-1—roots, stems, leaves, petals, receptacles, sepals, and ovules at 0, 1, 3, 5, 10, and 20 DPA and fibers at 10, 20, and 25 DPA [75]. The transcriptome data were aligned to the cotton genome using TopHat2 with default settings [76]. Cufflinks software was used to calculate the FPKM (fragments per kilobase of exon model per million mapped reads) value for each gene. Expression levels of *GhSMXL* genes in nine tissues were represented by log-transformed FPKM (log_2_[FPKM + 1]) values, and the R package pheatmap was used to visualize data.

Total RNA for each sample was isolated using a FastPure Plant Total RNA Isolation Kit (Polysaccharides & Polyphenolics–rich) (Vazyme, Nanjing) according to the manufacturer’s guidance. Total RNA was reverse transcribed into cDNA using a HiScript III RT SuperMix for qRT-PCR (Vazyme, Nanjing) kit. qRT-PCR was performed using a ChamQ Universal SYBR qPCR (Vazyme, Nanjing) kit on an Applied Biosystems 7500 Fast Real-Time PCR System (Life Technologies, Foster City, CA, USA). Gene-specific primers were designed using the online primer3 software (https://bioinfo.ut.ee/primer3-0.4.0/) (Table S8). qRT-PCR conditions were as previously described [77], with an initial denaturation step at 95 °C for 20 s, followed by 40 cycles of denaturation at 95 °C for 3 s and annealing/extension at 60 °C for 30 s. *GhUBQ7* was used as a reference gene [77]. Three independent qRT-PCR experiments were carried out, each with three mechanical repeats, and relative expression levels were determined using the 2^–∆Ct^ method [78].

### Protein co-expression network analysis

The ccNET database (http://structuralbiology.cau.edu.cn/gossypium) was utilized, which integrates the cotton genome, transcriptome, epigenome, and functional annotations. Protein sequences of SMXL6, SMXL7-1, and SMXL7-2 from *G*. *hirsutum* and *G*. *arboreum* were submitted to the website to compare the co-expression networks of SMXL proteins in diploid and tetraploid cottons. Key target proteins of SMXL6, SMXL7-1, and SMXL7-2 were identified, and functional annotations were performed.

### VIGS

For VIGS assays, approximately 200-bp nucleotide sequences of the open reading frames of *SMAX1-1* and *SMAX1-2* genes were amplified by PCR using gene-specific primers designed using the SGN VIGS Tool online software (https://vigs.solgenomics.net/) [79] (Table S8). The fragments obtained were then inserted into the pTRV2 vector to create pTRV2: *GhSMXL* constructs. The VIGS experiment was performed as described by Liu et al. (2021). Each pTRV: *GhSMXL* plasmid was introduced into *Agrobacterium tumefaciens* strain GV3101 and infiltrated into the cotyledons of 2-week-old *G*. *hirsutum* L. cv. ‘XLZ 33’ [80].

### Supplementary Information

**Additional file 1:** **Supplementary table S1.** Identification of SMXL gene family members from 21 plants. **Supplementary table S2.** Information of SMXL gene family in cotton (Gossypium sp.). **Supplementary table S3.** Amino acid sequences of motif 1-15 of GhSMXL proteins. **Supplementary table S4.** Information of the homologus·SMXL proteins in cotton (Gossypium sp.). **Supplementary table S5.** Syntenic SMXL gene pairs between allotetraploid and diploid cottons. **Supplementary table S6.** Analysis of network of SMXL6, 7-1, and 7-2 proteins in G.hirsutum and G.arboreum. **Supplementary table S7.** Information of the cotton database versions and websites used in this study. **Supplementary table S8.** Primer sequence information used in this study.

**Additional file 2:** **Fig. S1.** Multi nucleotide sequence alignment of GhSMXL genes. **Fig. S2.** Chromosomal distributions of SMXL genes in Gossypium spp. **Fig. S3.** Collinearity analysis of G. barbadense (At and Dt) orthologs in the genomes G. raimondii, G. arboreum, and G. herbaceum. **Fig. S4.** Expression characteristics of nine GhSMXL genes in 13 tissues determined using qRT-PCR. **Fig. S5.** Plant heights of the GhSMAX1-1 and GhSMAX1-2 silenced plants. **Fig. S6.** Information of cis-acting elements of GhSMXL genes.

## Data Availability

All data sheets and codes to process data are available upon request to the corresponding author, Xianzhong Huang (Huangxz@ahstu.edu.cn). The datasets generated and/or analysed during the current study are available in the NCBI Sequence Read Archive under BioProject ID: PRJNA490626.

## Abbreviations

SMXL: SUPPRESSOR OF MAX2 1 (SMAX1)-like
SLs: Strigolactones
EAR: Ethylene response factor-associated amphiphilic repression
SCF: Skp-Cullin-F-box
MAX2: More axillary branching
D14: DWARF14
KAI2: KARRIKIN INSENSITIVE2
TPR2: TOPLESS-RELATED PROTEIN2
MYA: Million years ago
WGD: Whole-genome duplication
WGT: Whole-genome triplication
qRT-PCR: Quantitative real-time PCR
DPA: Day post-anthesis
*K*a: Non-synonymous
*K*s: Synonymous
FPKM: Fragments per kilobase of exon model per million mapped reads
VIGS: Virus-induced gene silencing
