# Supplementary material for: Diversification of plant SUPPRESSOR OF MAX2 1 (SMAX1)-like genes and genome-wide identification and characterization of cotton SMXL gene family
Source: BMC Plant Biol. 2023 Sep 11;23:419. doi: 10.1186/s12870-023-04421-6 (PMC10494346; doi:10.1186/s12870-023-04421-6)
Supplement: Supplementary file 2 — Additional file 2: Fig. S1. Multi nucleotide sequence alignment of GhSMXL genes. Fig. S2. Chromosomal distributions of SMXL genes in Gossypium spp. Fig. S3. Collinearity analysis of G. barbadense (At and Dt) orthologs in the genomes G. raimondii, G. arboreum, and G. herbaceum. Fig. S4. Expression characteristics of nine GhSMXL genes in 13 tissues determined using qRT-PCR. Fig. S5. Plant heights of the GhSMAX1-1 and GhSMAX1-2 silenced plants. Fig. S6. Information of cis-acting elements of GhSMXL genes. [file 12870_2023_4421_MOESM2_ESM.docx]

## **Supplementary figure**


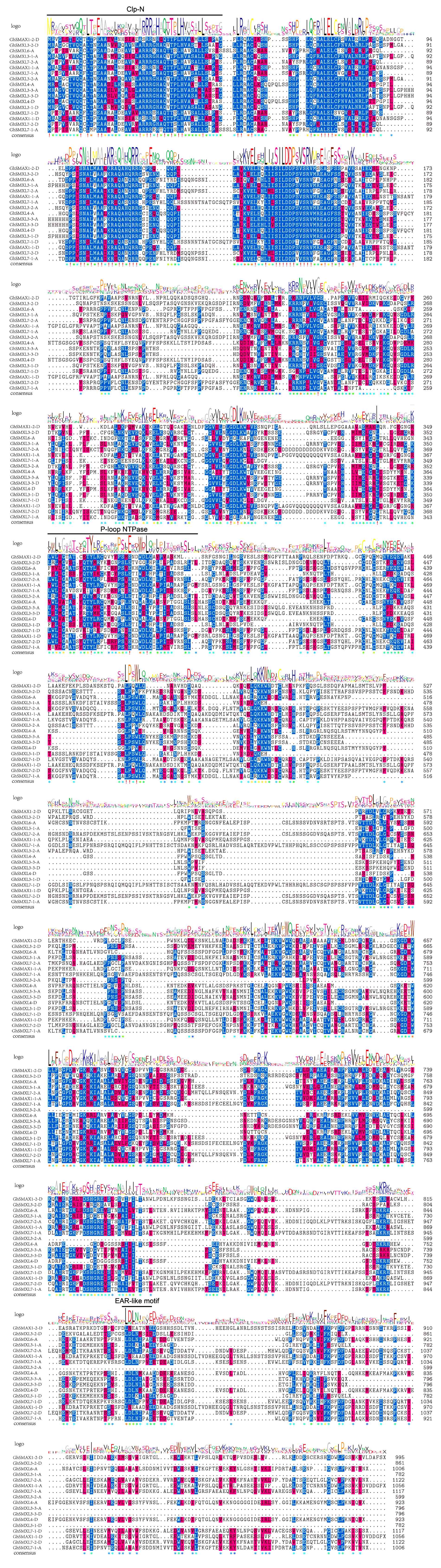


**Fig. S1** Multi nucleotide sequence alignment of *GhSMXL* genes.


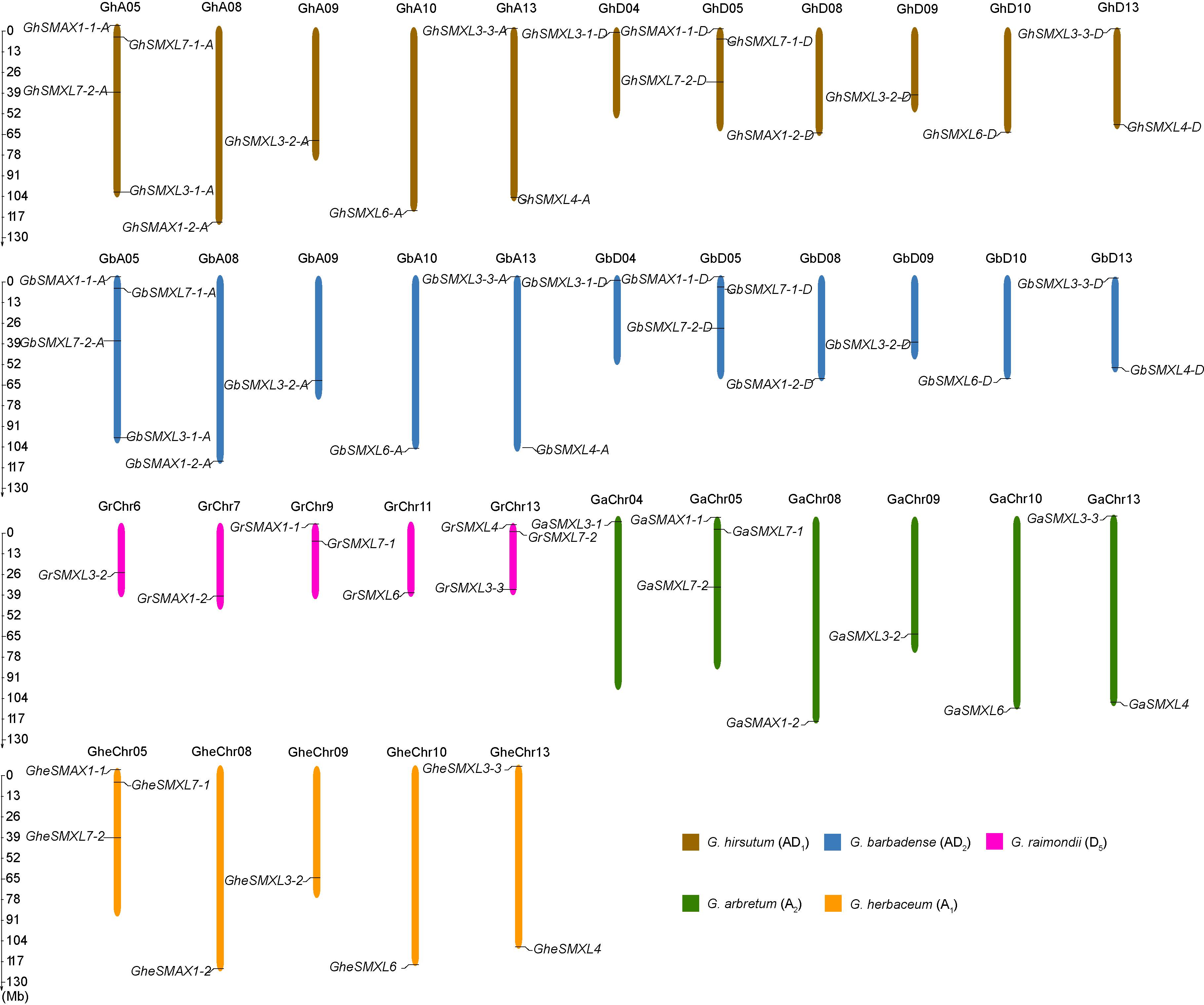


**Fig. S2** Chromosomal distributions of *SMXL* genes in *Gossypium* spp.
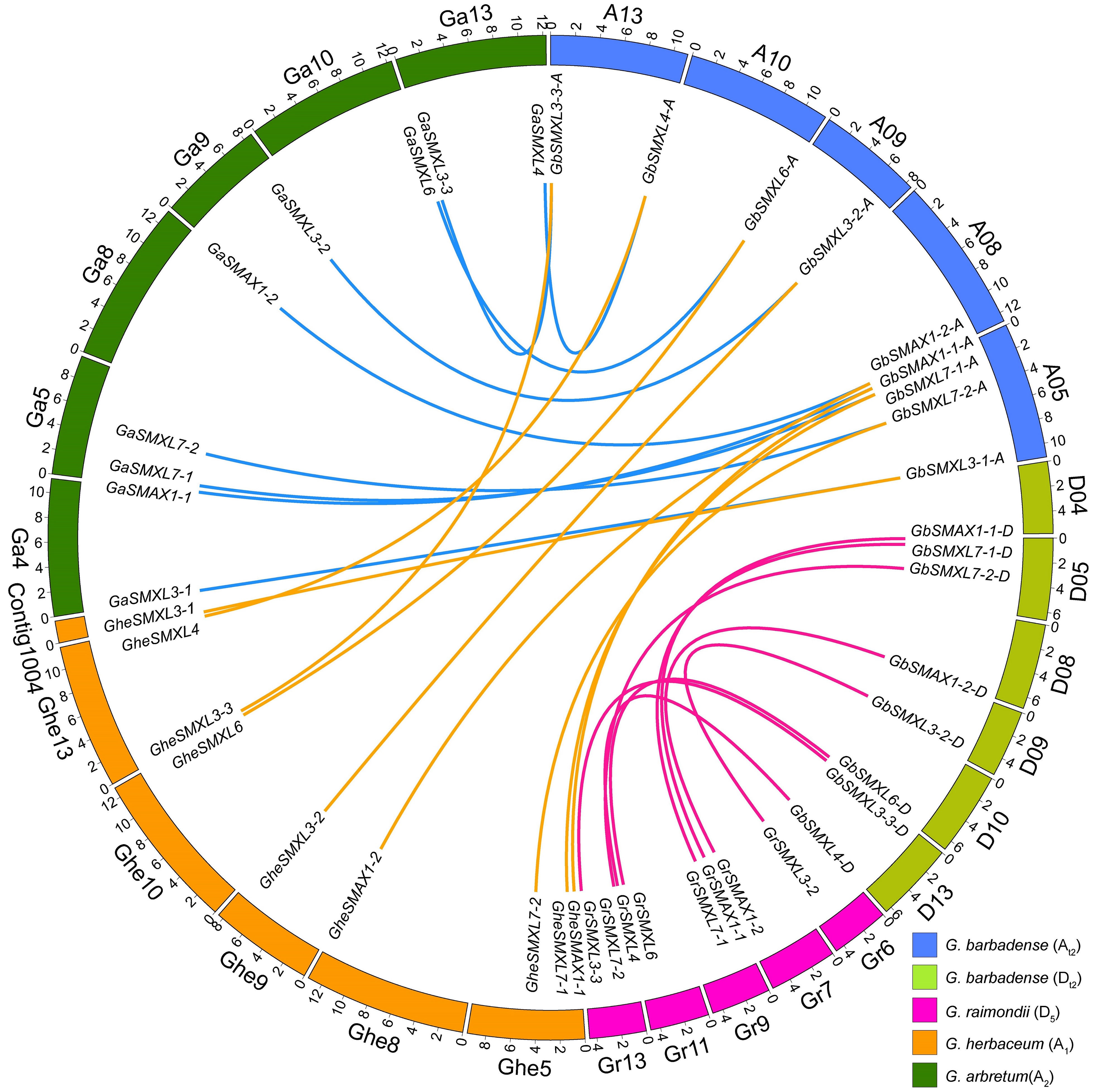


**Fig. S3** Collinearity analysis of *G. barbadense* (A_t_ and D_t_) orthologs in the genomes *G. raimondii*, *G. arboreum*, and *G. herbaceum*.

**
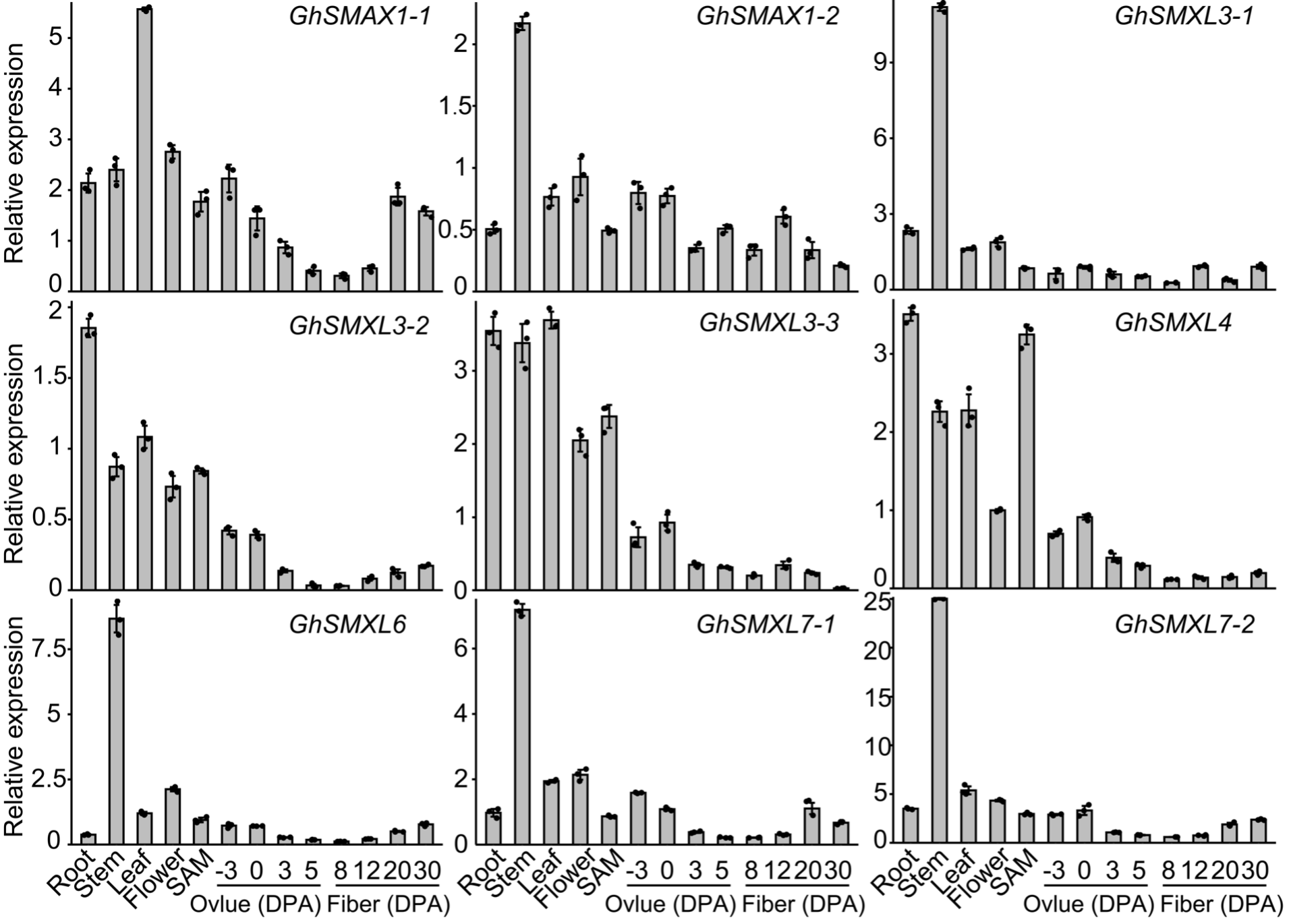
**

**Fig. S4** Expression characteristics of nine *GhSMXL* genes in 13 tissues determined using qRT-PCR.
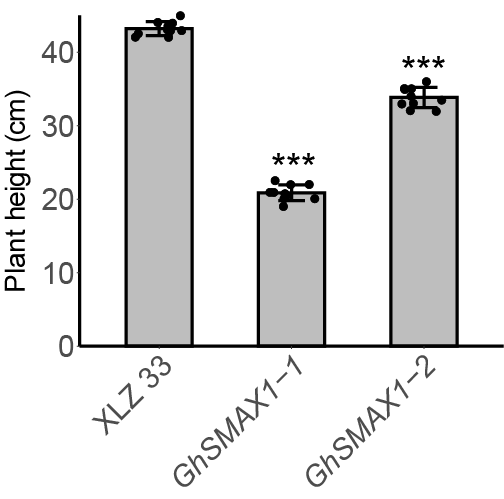


**Fig. S5** Plant heights of the *GhSMAX1-1* and *GhSMAX1-2* silenced plants.
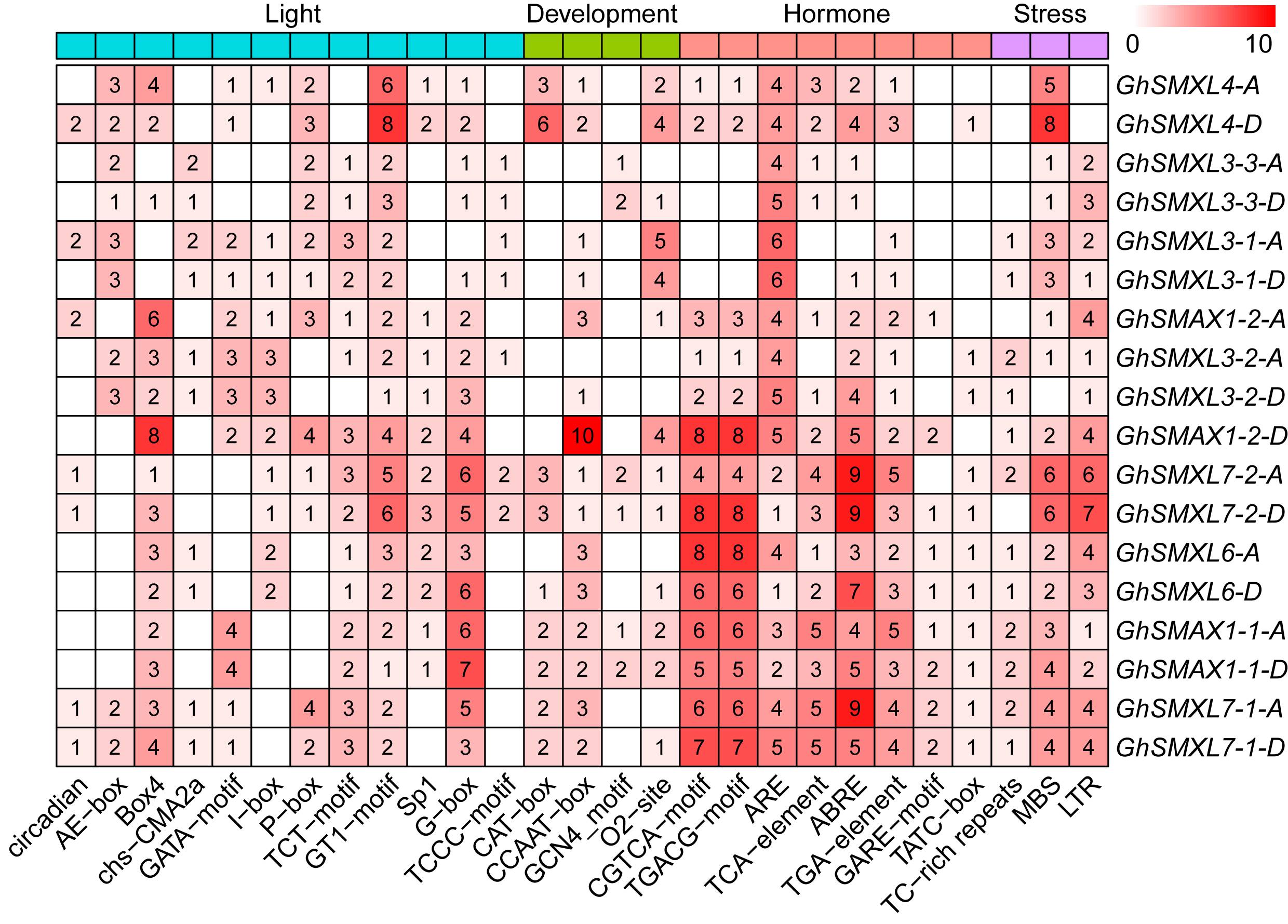


**Fig. S6** Information of cis-acting elements of *GhSMXL* genes.
